# Complete sequence of mitochondrial genome of *Cucumis melo* L.

**DOI:** 10.1080/23802359.2020.1808543

**Published:** 2020-08-17

**Authors:** Zhuo Ding, Haonan Cui, Qianglong Zhu, Yue Wu, Taifeng Zhang, Boyan Qiu, Peng Gao

**Affiliations:** a College of Horticulture and Landscape Architecture, Northeast Agricultural University, Harbin, Heilongjiang, China; b Ministry of Agriculture, Key Laboratory of Biology and Genetic Improvement of Horticulture Crops (Northeast Region), Harbin, Heilongjiang, China; c Department of Horticulture, College of Agronomy, Jiangxi Agricultural University, Nanchang, P.R. China

**Keywords:** *Cucumis melo* L., mitochondrial genome

## Abstract

*Cucumis melo* L. is one of the most important fruit-type vegetables in the world. This genome is divided into a main loop and two small loops. The length of the main loop is 2,709,526 bp, and the two small loops are 149,555 bp and 47,592 bp long, respectively. There are 88 coding genes in the melon mitochondrial genome, including 40 protein-coding genes (which accounted for about 1.23% of the whole genome), 8 rRNAs, and 40 tRNAs. The total length of rRNAs and tRNAs spans 0.31% of the total genome sequence. Among the 88 mitochondrial coding genes, only 5 tRNAs were located into the second largest circular DNA molecule. The complete mitogenome sequence provided herein would help understand *C. melo* evolution.

*Cucumis melo* L. is a Cucurbitaceae crop and one of the most important fruit-type vegetables in the world. Mitochondria is an important organelle in plant cells, also known as the ‘energy factory’ of cells which contain independent genetic materials and systems. These additional mitochondrial genomes have increased our understanding of genome rearrangement, DNA transfer, and phylogenetic diversity. Plant mitochondrial genomes encode tRNAs, rRNAs, proteins and ribosomal proteins, and range in size from 200 Kb in *Brassica hirta* (Palmer and Herbon, 1987) to 1.33 Mb in *Hevea brasiliensis* (Shearman et al. 2014).

Sample of *C. melo* (accession number: MR-1) was stored in the College of Horticulture of Northeast Agricultural University (126°43′16.7″E, 45°44′23.8″N), Harbin, China. Mitochondrial DNA was extracted according to a slightly modified version of previous methodologies, as described by Shen Jia (Sheng 2015) and Rodriguez-Moreno (Rodríguez-Moreno et al. 2011). The mitochondrial DNA samples were sequenced using a PacBio Sequel by Beijing Genomics Institution. A total of 4.14 Gb of the original data was obtained by PacBio Sequel sequencing. A total of 3.94 Gb of sequenced data was obtained after quality control analysis. The range of reading length for long DNA fragments was between 50 and 63,847 bp, with an average length is 6978 bp and GC content of 44.1%. This genome is divided into a main loop and two small loops. The length of the main loop is 2,709,526 bp, and the two small loops are 149,555 bp and 47,592 bp long, respectively. The annotated melon mitochondrial genome has been submitted to NCBI/GenBank (Accession numbers MG947207 to MG947209).

We have found 88 coding genes in the melon mitochondrial genome, including 40 protein-coding genes (which accounted for about 1.23% of the whole genome), 8 rRNAs and 40 tRNAs. The total length of rRNAs and tRNAs spans 0.31% of the total genome sequence. Among the 88 mitochondrial coding genes, only 5 tRNAs were located into the second largest circular DNA molecule.

We performed a phylogenetic analysis of mitochondrial protein-coding genes in melon, watermelon, cucumber and zucchini. For the analysis of phylogenetic relationship between cucurbitaceae and grape based on comparative nuclear genomics (Urasaki et al. 2017; Wu et al. 2017; Wang et al. 2018), we included the mitochondrial protein-coding genes of grape (*Vitis vinifera*, Gen Bank NO.：NC_012119.1) as a non-related group (control). Mitochondrial genomes were aligned using ClustalX (http://www.clustal.org/clustal2/). A phylogenetic tree was constructed via Bayesian inference (BI) approaches using Beast software (Suchard et al. 2018). Based on these sequences, the evolutionary relationships of the four Cucurbitaceae crop systems were examined (Figure 1). A phylogenetic tree, corresponding to respective plant mitochondrial genomes, indicate that melon has a more recent evolutionary relationship with cucumber, and watermelon has a more recent evolutionary relationship with zucchini. In this mitochondrial gene tree, melon and cucumber crops were separated from watermelon, and the time of independent evolution was estimated to be ∼14.69 million years. The complete mitogenome sequence provided herein would help understand *C. melo* evolution.

**Figure 1.**
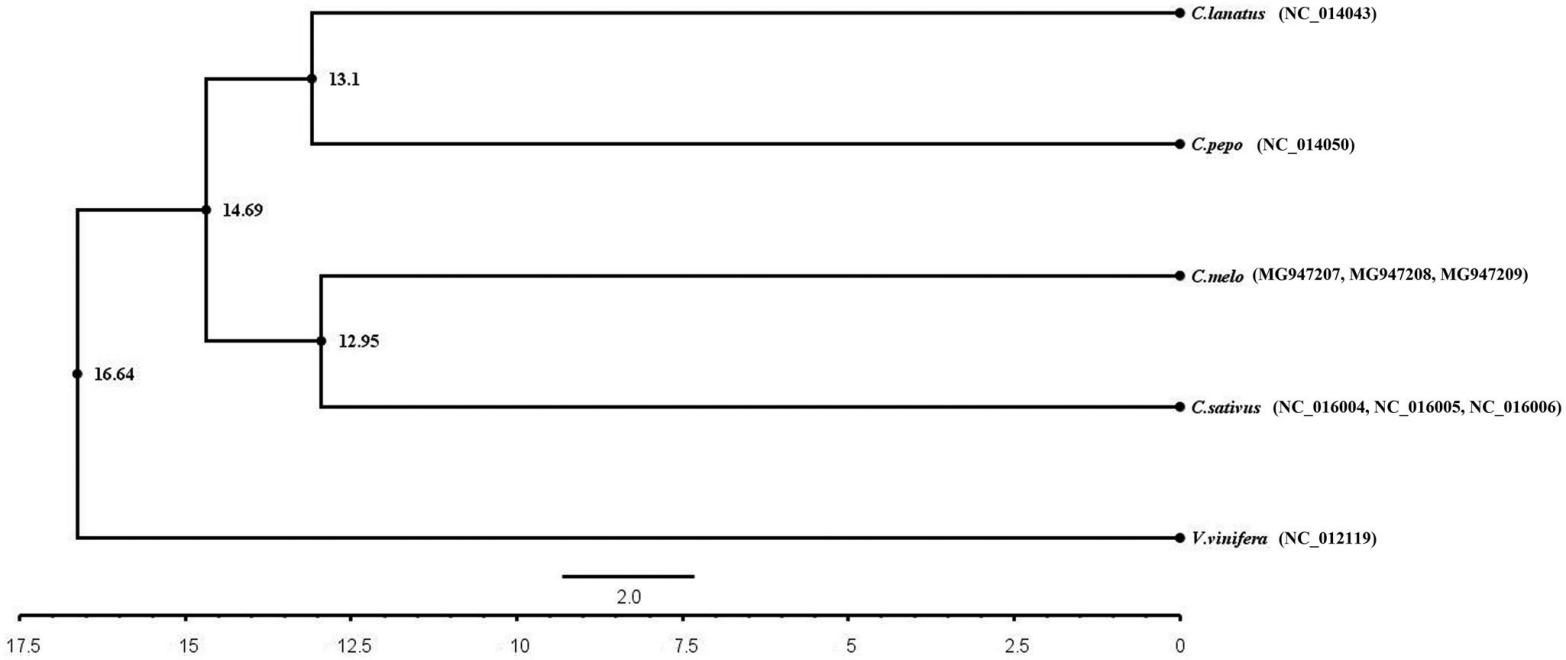
The construction of phylogenetic tree among 4 cucurbit plants based on mitochondrial protein-coding gene.

## Data Availability

The data that support the findings of this study are openly available in GenBank of NCBI at https://www.ncbi.nlm.nih.gov, reference numbers: MG947207; MG947208; MG947209.
